# On the role of longitudinal currents in radiating systems of charges

**DOI:** 10.1038/s41598-024-66848-7

**Published:** 2024-07-19

**Authors:** Nikita A. Nemkov, Vassili A. Fedotov

**Affiliations:** 1 Russian Quantum Center, Skolkovo, Moscow, Russia 121205; 2 National University of Science and Technology MISIS, Moscow, Russia 119049; 3 Centre for Photonic Science and Engineering, Skolkovo Institute of Science and Technology, Moscow, Russia 121125

**Keywords:** Condensed-matter physics, Optical physics

## Abstract

The time derivative of a charge density is linked to a current density by the continuity equation. However, it features only the longitudinal part of a current density, which is known to produce no radiation. This fact usually remains unnoticed and may appear puzzling at first, suggesting that the temporal variation of a charge density should be also irrelevant to radiation. We alleviate the apparent contradiction by showing that the effective longitudinal currents are not spatially confined, even when the time-dependent radiating charge density that generates them is. This enforces the co-existence of the complementary, i.e. transverse, part of the current, which, in turn, gives rise to radiation. We illustrate the necessarily delocalized nature and relevance of longitudinal currents to the emission of electromagnetic waves by a dynamic electric dipole, discussing the practical implications of that for radation in partially conducting condensed matter. More generally, we show how the connection between the longitudinal and transverse currents shapes the structure of the conventional multipole expansion and fuels the ongoing confusion surrounding the charge and toroidal multipoles.

## Introduction

With the current surge of interest in non-radiating electromagnetic modes in condensed matter, such as dynamic anapoles and bound states in the continuum found in photonic crystals, metamaterials and nanostructured materials (see, for example^1–11^, and references therein), it has also become important to revisit and address certain peculiarities of their counterparts, i.e., radiating modes.

In particular, in many problems of classical electrodynamics involving electromagnetic radiation it is methodologically useful to substitute a time-dependent charge density $\rho (t)$, with its current density equivalent $\varvec{j}(t)$, related by the continuity equation (see, for example^12^),

1 $$\begin{aligned} \partial _t\rho +\varvec{\nabla }\cdot \varvec{j}=0 \ . \end{aligned}$$

In this article the term current density and notation $\varvec{j}$ are always related to conduction currents. We do not introduce displacement currents and discuss the properties of (the derivative) of the electric field directly, when appropriate.

However, this generally adopted approach leads to the following puzzling observation which, to the best of our knowledge, has not been properly discussed in textbooks on electrodynamics. On the one hand, the time-dependent charge density gives rise to radiation—accelerating particles and oscillating electric dipoles are the well-known examples here. On the other hand, the continuity equation is missing (though implicitly) the transverse part of the current density $\varvec{j}_\perp$, which is held responsible for generating radiation fields, i.e. electric and magnetic fields outside the volume occupied by the charge density, see e.g. classic paper^13^ or textbook^14^. Indeed, assuming that the vector field $\varvec{j}(r,t)$ is sufficiently smooth and decays sufficiently fast at infinity, it can be split via the Helmholtz decomposition into the longitudinal ($\varvec{\nabla }\times \varvec{j}_\parallel = 0$) and transverse ($\varvec{\nabla }\cdot \varvec{j}_\perp = 0$) components, and so in the continuity equation $\varvec{\nabla }\cdot \varvec{j} = \varvec{\nabla }\cdot (\varvec{j}_\parallel + \varvec{j}_\perp ) = \varvec{\nabla }\cdot \varvec{j}_\parallel$. Since longitudinal currents do not radiate, the time-dependent charge density must also be irrelevant for the radiation.

To solve this seeming puzzle one needs to accept that the longitudinal and transverse parts of a spatially localized current density are not completely independent, as was briefly noted in^15^. Moreover, even when non-stationary charges in a condensed matter system are confined to some finite region of space, the transverse and longitudinal components of a current density not only can exist outside that region but extend to infinity (provided that $\varvec{j}_\parallel = -\varvec{j}_\perp$ there^16^). This simple yet, perhaps, counterintuitive observation, made in^12^ only in passing, is one of the main points that we elaborate on in this paper.

## Longitudinal and transverse currents of a localized source

We begin by clarifying how (or rather when) a vector field can be simultaneously transverse and longitudinal. One usually identifies a vector field with such a property while deriving the Helmholtz decomposition, but here we are going to avoid the lengthy derivation and offer a brief and perhaps simpler alternative in the style of^17^.

If a vector field $\varvec{j}$ (which at the moment is not necessarily a current density) is longitudinal, i.e. $\varvec{\nabla }\times \varvec{j}=0$, it can always be represented as the gradient of some scalar potential $\psi$

2 $$\begin{aligned} \varvec{j}=\varvec{\nabla }\psi \ . \end{aligned}$$

The requirement for this field to be simultaneously transverse, i.e. $\varvec{\nabla }\cdot \varvec{j} = 0$, leads to $\nabla ^2\psi = 0$, where $\nabla ^2$ is the scalar Laplacian. If this condition holds everywhere in space the corresponding current is vanishing (assuming that $\varvec{j}$ decays sufficiently fast at spatial infinity it follows $\int d^3\varvec{r} \,\,\varvec{j}(\varvec{r})\cdot \varvec{j}(\varvec{r})=-\int d^3\varvec{r}\,\, \psi \nabla ^2\psi =0$ and hence $\varvec{j}(\varvec{r})=0$ everywhere). However, if  $\nabla ^2\psi = 0$ holds only outside some region *R*, then $\psi$ can be perfectly non-trivial, and so $\varvec{j}$ will be both transverse and longitudinal outside *R*.

In fact, this type of fields is not at all exotic. The electric field of (almost) any static distribution of charges is of this kind. Indeed, consider a static charge density $\rho _s$ confined to *R*. Its scalar potential $\phi _s$ is governed by the Poisson equation

3 $$\begin{aligned} \nabla ^2\phi _s = -\frac{\rho _s}{\varepsilon _0} \ , \end{aligned}$$

where we introduce subscript *s* to indicate quantities related to this auxiliary static problem.

Outside *R* such a potential naturally satisfies $\nabla ^2\phi _s = 0$ and, therefore, the electric field generated there $\varvec{E}_s=-\varvec{\nabla }\phi _s$ is simultaneously longitudinal and transverse. Importantly, this general example also shows that the leakage of the longitudinal field component outside the confined source and up to infinity is almost inevitable (there are exceptions though, as discussed in Supplementary Information, Appendix B).

Now let us consider a general current $\varvec{j}$ with its Helmholtz decomposition into transverse and longitudinal parts, $\varvec{j}=\varvec{j}_\parallel +\varvec{j}_\perp$. We will show that both $\varvec{j}_\parallel$ and $\varvec{j}_\perp$ generically extend to infinity even when $\varvec{j}$ is confined. Scalar potential $\psi$ that defines the longitudinal part $\varvec{j}_\parallel =\varvec{\nabla }\psi$ can be found from condition $\nabla ^2\psi =\varvec{\nabla }\cdot \varvec{j}$ or

4 $$\begin{aligned} \nabla ^2\psi = -\partial _t \rho \ , \end{aligned}$$

where we have used the continuity equation Eq. (1). This is mathematically the same problem as Eq. (3) with $\psi$ in place of $\phi _s$ and $\partial _t\rho$ in place of $\rho _s/\varepsilon _0$. This means that $\varvec{j}_\parallel$ is proportional to the time-derivative of the longitudinal part of the electric field produced by the actual charge density

5 $$\begin{aligned} \varvec{j}_\parallel =-\varepsilon _0\partial _t \varvec{E}_\parallel \ . \end{aligned}$$

One could have derived this result directly from the Maxwell equation for the current density

6 $$\begin{aligned} \varvec{j}=-\varepsilon _0\partial _t\varvec{E}+\frac{1}{\mu _0}\varvec{\nabla }\times \varvec{H} \end{aligned}$$

by considering its longitudinal component. However, it would then be unclear that $\varvec{j}_\parallel$ was not simply vanishing. Indeed, one is naturally tempted to conclude that $\varvec{E}_\parallel = 0$ because the electric field is transverse in the radiation zone (i.e. $\varvec{\nabla }\cdot \varvec{E} = 0$). This conclusion is obviously premature since $\varvec{E}_\parallel$ and $\varvec{E}_\perp$ must be defined everywhere in space (and not only in the radiation zone), and hence $\varvec{E}_\parallel$ is (typically) non-zero.

As we have demonstrated above, the longitudinal electric field usually extends beyond the confines of a static distribution of charges and, thus, the longitudinal component of the current density $\varvec{j}_\parallel$ does the same in the dynamic case. The most important consequence of the spatially delocalized nature of $\varvec{j}_\parallel$ is that the transverse component of the current density $\varvec{j}_\perp$ must also be non-vanishing, even though this cannot be inferred directly from the continuity equation. This simply follows from the fact that the total current density $\varvec{j}=\varvec{j}_\parallel +\varvec{j}_\perp$ vanishes outside the source and hence

7 $$\begin{aligned} \varvec{j}_\perp =-\varvec{j}_\parallel \qquad (\hbox { outside}\ R) \ . \end{aligned}$$

Now we are ready to revisit the seeming puzzle posed in the introduction. Given that $\varvec{j}_\parallel$ and $\varvec{j}_\perp$ of a localised source are linked, a more precise statement is not that the longitudinal currents are irrelevant to the radiation, but rather that the radiation fields can be expressed solely via the transverse currents. Indeed, a vector potential $\varvec{A}$ can be written in the following form (see Supplementary Information, Appendix A)

8a $$\begin{aligned} \varvec{A}(\varvec{r})=-\frac{\mu _0}{k^2}\varvec{j}_\parallel (\varvec{r})+\frac{\mu _0}{4\pi }\int _R d\varvec{r}'\,G(\varvec{r}-\varvec{r}')\varvec{j}_\perp (\varvec{r'}) \ . \end{aligned}$$

Here $\varvec{A}$ is non-locally related to $\varvec{j}_\perp$, i.e. the distribution of $\varvec{j}_\perp$ inside *R* crucially affects the radiation field outside *R*. However, the longitudinal part of the current density enters $\varvec{A}$ only locally. Since Eq. (8a) holds in any region of space, one can replace $\varvec{j}_\parallel$ with $- \varvec{j}_\perp$ outside *R* and, as a result, the radiation fields will be expressed solely via $\varvec{j}_\perp$

8b $$\begin{aligned} \varvec{A}(\varvec{r})=\frac{\mu _0}{k^2}\varvec{j}_\perp (\varvec{r})+\frac{\mu _0}{4\pi }\int _R d\varvec{r}'\,G(\varvec{r}-\varvec{r}')\varvec{j}_\perp (\varvec{r'}) \qquad (\hbox { outside}\ R) \ . \end{aligned}$$

At the same time, one usually cannot alter $\varvec{j}_\perp$ without altering $\varvec{j}_\parallel$ (or, equivalently, the charge density of the source) and so it would be misleading to state that $\varvec{j}_\parallel$ is completely irrelevant to the radiation. This is especially true if the radiation occurs in partially conducting condensed matter (such as sea water), where the total current density outside the source does not have to be zero. We consider this case in more detail in the next section.

## Electric dipole

The preceding discussion was rather abstract and we would now like to illustrate it using the simplest non-trivial example of a radiating localized source—a non-stationary electric dipole of negligible size. For the dipole moment $\varvec{d}$ placed at the origin $r=0$ the charge and current densities are (we denote $\partial _t\varvec{d}=\dot{\varvec{d}}$)

9 $$\begin{aligned} \rho = -\varvec{d} \cdot \varvec{\nabla } \,\,\delta (\varvec{r}) ,\qquad \varvec{j}= \dot{\varvec{d}}\,\delta (\varvec{r}) \ . \end{aligned}$$

Neither $\varvec{\nabla }\times \varvec{j}$ nor $\varvec{\nabla }\cdot \varvec{j}$ vanish everywhere, so $\varvec{j}$ contains both longitudinal and transverse parts. Let us explicitly construct $\varvec{j}_\parallel$ corresponding to the point dipole. Since $\varvec{\nabla }\cdot \varvec{j}_\perp =0$  by definition and $\varvec{j}_\parallel =\nabla \psi$ for some $\psi$, Eq. (1) transforms into

10 $$\begin{aligned} \nabla ^2\psi = \dot{\varvec{d}} \cdot \varvec{\nabla }\delta (\varvec{r}) \ , \end{aligned}$$

which has the following solution

11 $$\begin{aligned} \psi = -\dot{\varvec{d}} \cdot \varvec{\nabla }\frac{1}{4\pi r} \ , \end{aligned}$$

due to the well-known identity $\delta (\varvec{r})=-\nabla ^2\frac{1}{4\pi r}$. By construction, the longitudinal part of the corresponding current density is given by the gradient of $\psi$

12 $$\begin{aligned} \varvec{j}_\parallel = \varvec{\nabla }\psi = -\dot{\varvec{d}} \cdot \varvec{\nabla }\left( \varvec{\nabla }\frac{1}{4\pi r}\right) =\frac{\dot{\varvec{d}}-3(\dot{\varvec{d}} \cdot \varvec{n})\varvec{n}}{4\pi r^3} +\frac{1}{3}\dot{\varvec{d}}\delta (\varvec{r})\ , \end{aligned}$$

where $\varvec{n}=\varvec{r}/r$. At any given moment this is nothing but the electric field produced by the static electric dipole with the moment $\varvec{d}_s=-\epsilon _0\partial _t\varvec{d}$. We emphasize that Eq. (12) makes it evident that $\varvec{j}_\parallel$ extends beyond $r=0$, and, in fact, up to infinity. Also, a care is needed when taking second derivatives of $\frac{1}{r}$. We used equation $\nabla _i\nabla _j\frac{1}{r}=\frac{3n_in_j-\delta _{ij}}{r^3}-\frac{4\pi }{3}\delta ^{ij}\delta (\varvec{r})$, see Ref.^18^. This equation comes with a certain regularization prescription near $r=0$, a subtlety not relevant for our discussion, which is focused on the behavior of $\varvec{j}_\parallel$ and $\varvec{j}_\perp$ away from the source. Alternatively, simply compare Eq. (12) with the electric field of the point electric dipole, see Eq. (3.106) in^17^. Of course, for time-dependent $\varvec{d}$ the “electric field“ in Eq. (12) is time-dependent as well, but at each moment in time it has the shape of the electric field of a static dipole. Finally, we stress that $\dot{\varvec{d}}(t)$ is evaluated at the present, not at the retarded time.

If the electric dipole resides in vacuum, $\varvec{j}_\parallel$ must be canceled outside $r = 0$, so that the total current density produced externally will remain vanishing. This is possible only by admitting the co-existence of the spatially non-localized complementary, transverse part of the current density, which takes the following form in the case at hand

13 $$\begin{aligned} \varvec{j}_\perp = \varvec{\nabla }\times \varvec{\nabla }\times \frac{\dot{\varvec{d}}}{4\pi r} \ . \end{aligned}$$

Indeed

14 $$\begin{aligned} \varvec{j}_\perp =\left( (\dot{\varvec{d}}\cdot \varvec{\nabla }) \varvec{\nabla }-\dot{\varvec{d}}\,\nabla ^2\right) \frac{1}{4\pi r}=(\dot{\varvec{d}}\cdot \varvec{\nabla }) \varvec{\nabla }\frac{1}{4\pi r}+\dot{\varvec{d}}\,\delta (\varvec{r}) \ . \end{aligned}$$

The form of transverse current (13) can be verified by direct substitution, as we do in the main text, or, more formally, derived as a solution of the differential equation $\varvec{j}_\perp =\varvec{\nabla }\times \varvec{v}$ for a vector field $\varvec{v}$ that turns out to be $\varvec{v}=\varvec{\nabla }\times \frac{\dot{\varvec{d}}}{4\pi r}$.

The first term in Eq. (14) is equal to $-\varvec{j}_\parallel$, while the second term gives the spatially localized current density of the point electric dipole (9).

**Figure 1:**
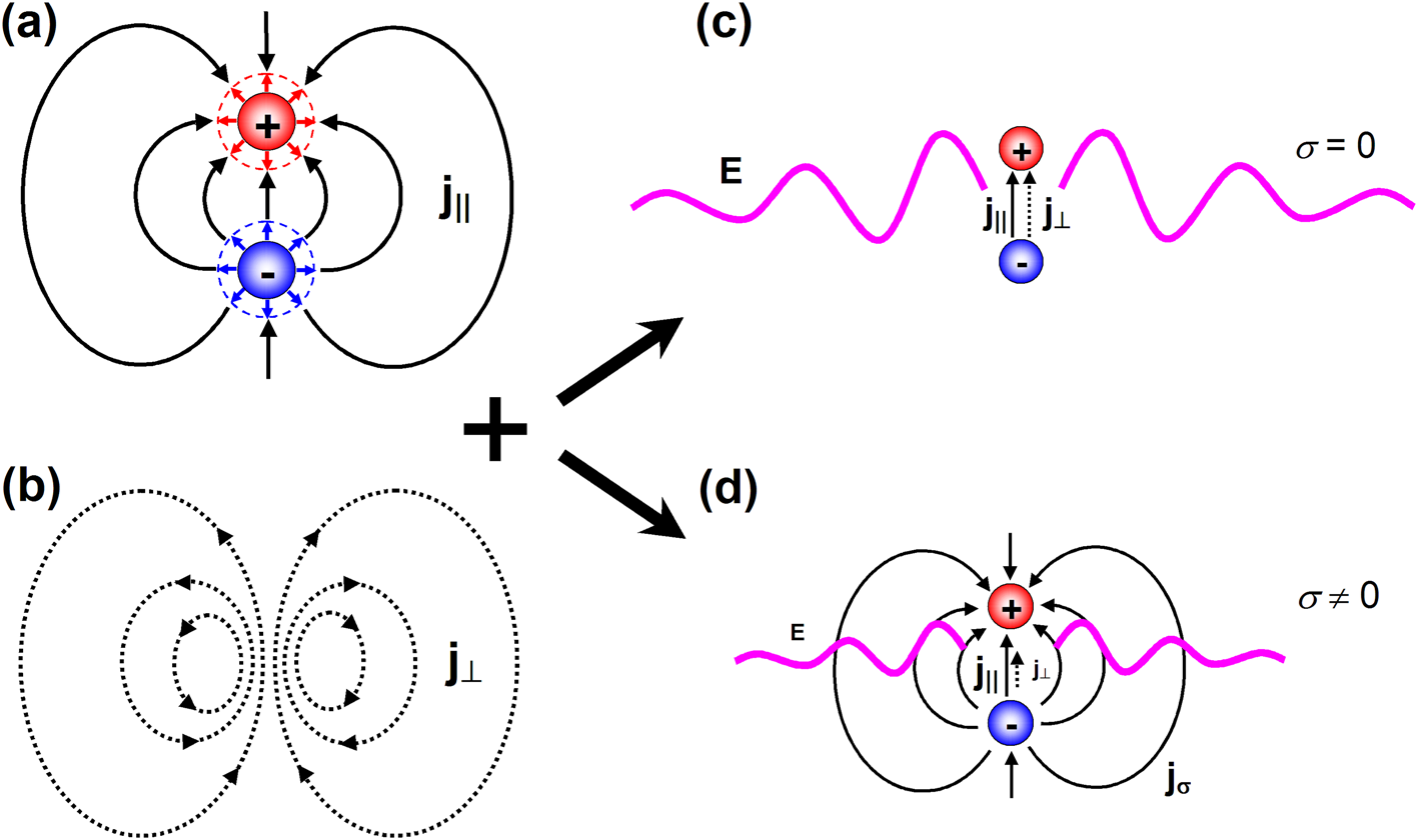
(**a**) An oscillating electric dipole shown schematically as a pair of non-stationary charge distributions of opposite signs at a moment in time when the charge densities increase (as illustrated by outward pointing red/blue arrows surrounding the charges). Curved black arrows represent longitudinal currents, which ensure the change in accordance with the continuity equation. (**b**) The distribution of transverse currents, which can counteract longitudinal currents beyond the confines of the electric dipole. (**c**) The electric dipole radiates in non-conducting medium, where the cancellation of longitudinal currents by transverse currents must be complete. (**d**) An electric dipole radiates less efficiently in partially conducting medium since the complete cancellation of longitudinal currents is not required, and so transverse currents (responsible for radiation) are weaker.

Figure 1 offers a graphical illustration of the above analysis. Without loss of generality we assume that the radiating electric dipole is formed by a pair of spatially localized positive and negative charge distributions (see Fig. 1a). We further assume that the change of the electric dipole moment occurs due to oscillations of the charge densities (rather than the distance between the two).

We now consider a moment in time when the charge densities increase. According to the continuity equation, an increase in charge density is ensured by currents flowing in (or out) of the volume containing the charges, and those currents are longitudinal. In our case the currents must flow towards the positive charge distribution, where positive charges are accumulated, and away from the negative charge distribution, where positive charges are lost. Considering each charge distribution separately one can already appreciate the fact that the longitudinal currents will formally extend to infinity.

In a physical system longitudinal currents must find their source and drain (due to charge conservation). In our example this means that the currents leaving the negative charge distribution and entering positive charge distribution must be connected everywhere (including infinity), as illustrated in Fig. 1a. However, in a typical situation—when an electric dipole is located in a dielectric or vacuum—charges cannot be physically transported outside the confines of the dipole and, hence, the longitudinal currents must appear somehow negated.

At this point one has to admit the presence of complementary oscillating transverse currents (see Fig. 1b). Such currents require neither a source nor a drain, and “can be arranged“ in space in a way that they will cancel longitudinal currents everywhere apart from the central region, where they have the same density and flow in the same direction as the longitudinal currents. The resulting picture (Fig. 1c) is now intuitively more acceptable, since it shows the electric dipole as a spatially localised charge-current distribution, the radiation of which is allowed through the presence of confined transverse currents.

One may still wonder why going to the trouble of introducing into the picture spatially non-localized currents for an a priori localized electromagnetic source, especially that those currents cannot be sustained outside the source, and thus appear purely virtual. As we have pointed out earlier, such an approach enables one to show explicitly why a system of non-stationary charges acquires a transverse component of its real current density and, therefore, can radiate.

Furthermore, and perhaps more importantly, admitting spatially non-localized currents helps one to understand the peculiarities of antenna radiation in partially conducting condensed matter, such as sea water or soil^19,20^. Indeed, in such media the longitudinal currents outside the electric dipole in Fig. 1a can be partially sustained (via real conductivity) and, therefore, are not required to be completely negated by transverse currents. Correspondingly, the current balance outside the electric dipole (7) is modified as follows

15 $$\begin{aligned} \varvec{j}_\perp +\varvec{j}_\parallel =\varvec{j}_\sigma \ , \end{aligned}$$

where $\varvec{j}_\sigma$ is the density of the actual longitudinal currents supported by the medium due to its non-vanishing electric conductivity. Since $\varvec{j}_\parallel$ remains fixed by the continuity equation, $|\varvec{j}_\perp |<|\varvec{j}_\parallel |$ and this inequality becomes only stronger with increasing conductivity of the medium. As a result, not only the emitted electromagnetic waves will decay faster than in vacuum/air (due to absorption) but also the overall radiation efficiency of an electric dipole antenna will become lower even in a well-matched case (see Fig. 1d).

In plain words, the fraction of energy that would have been radiated by the antenna is now circulated in the medium (and dissipated) via real longitudinal currents. This, in particular, might explain anomalously high attenuation of radiation from electric dipole antennas in sea water and shows why loop (i.e., magnetic dipole) antennas, which do not feature longitudinal currents, are generally better emitters in partially conducting condensed matter^19,20^. Indeed, in the case of a point magnetic dipole, $\rho = 0$, $\varvec{\nabla }\cdot \varvec{j}=0$ and $\varvec{j}_\parallel =0$.

## Implications for the multipole expansion

Another way to formulate the puzzle that we have started this paper with is to note that in a typical radiating system in condensed matter the charge multipoles, such as an electric dipole or quadrupole, are central to radiation. Their moments, however, are defined solely by the charge density. For instance,

16 $$\begin{aligned}&\varvec{D}=\int d\varvec{r}\, \varvec{r} \rho (\varvec{r}) \ ,\end{aligned}$$

17 $$\begin{aligned}&Q_{\alpha \beta }=3\int d\varvec{r}\, \left( 3r_\alpha r_\beta -r^2\delta _{\alpha \beta }\right) \rho (\varvec{r}) \ , \end{aligned}$$

where $\varvec{D}$ and *Q* are the electric dipole and quadrupole moments, respectively. The charge density is formally related only to the longitudinal currents $\varvec{j}_\parallel$  of Eq. (1) and, therefore, it is seemingly irrelevant for the radiation fields, which can be defined only through transverse currents $\varvec{j}_\perp$. Now, of course, we appreciate that there is no contradiction, because $\varvec{j}_\parallel$ is spatially delocalized and, hence, intimately connected to $\varvec{j}_\perp$. In particular, the time-derivatives of all electric multipole moments can be expressed via $\varvec{j}_\perp$. For instance,

18 $$\begin{aligned} \partial _t\varvec{D}=\int d\varvec{r}\,\, \varvec{r} \partial _t\rho (\varvec{r})=-\int d\varvec{r}\,\, \varvec{j}=-\int d\varvec{r}\,\, \varvec{j}_\perp \ . \end{aligned}$$

Here we used the Helmholtz decomposition and the fact that $\int d\varvec{r} \, \varvec{j}_\parallel =0$, which is not entirely trivial but true, as shown in Supplementary Information, Appendix C.

It seems, however, that the delocalized character of longitudinal currents and their connection to the transverse currents brings about an additional confusion to the structure of the multipole expansion. The full multipole expansion of an arbitrary current density in a condensed matter system consists of the charge, magnetic and toroidal multipoles. For instance, magnetic and toroidal dipole moments are defined by^21^

19 $$\begin{aligned} \varvec{M}=\frac{1}{2} \int d\varvec{r}\,\, \varvec{r}\times \varvec{j} \ , \end{aligned}$$

20 $$\begin{aligned} T_\alpha =\frac{1}{10} \int d\varvec{r}\,\, \left( r_\alpha r_\beta -2r^2\delta _{\alpha \beta }\right) j_\beta \ . \end{aligned}$$

It is sometimes claimed that the toroidal multipole family represents merely higher-order corrections to the charge multipoles^22^. This is, of course, not true – the two families are independent of each other, just as they are independent of the magnetic multipoles, with one notable exception – the leading order.

To explain this we need to recall additional terms of the multipole expansion of a non-stationary charge-current distribution, the mean-square radii^21,23,24^. For any given multipole moment (which can be viewed as a measure of certain angular properties of the charge-current distribution) there is a series of the mean square radii, which characterizes the radial profile of the corresponding multipolar mode. For example, the mean-square radii of an electric dipole moment are defined by

21 $$\begin{aligned} \varvec{D}^{(n)}\propto \int d\varvec{r}\,\, r^{2n}\varvec{r}\rho (\varvec{r}) \end{aligned}$$

and one simply inserts an additional factor of $r^2$ to obtain $\varvec{D}^{(n+1)}$ from $\varvec{D}^{(n)}$. Here and further in this section $\propto$ means equality up to non-zero numerical factors, which we do not keep track of. One can also move in the reverse direction, i.e. from $\varvec{D}^{(n+1)}$ to $\varvec{D}^{(n)}$, by inserting $\nabla ^2\rho$ in place of $\rho$:

22 $$\begin{aligned} \varvec{D}^{(n)}\propto \int d\varvec{r}\,\,r^{2n+2}\varvec{r}\,\,\nabla ^2\rho (\varvec{r})\propto \int d\varvec{r}\,\,r^{2n}\varvec{r}\rho (\varvec{r}) \ . \end{aligned}$$

If one continuous the reverse transformation further, one will see that the lowest-order term in the series, i.e. the “parent“ term (given by $n = 0$) is, of course, the electric dipole moment itself, $\varvec{D}^{(0)}=\varvec{D}$.

The point we wish to make is that the same procedure applied to the mean-square radii of a toroidal dipole will also yield an electric dipole moment as the lowest-order term of the series, but now corresponding to $n = -1$

23 $$\begin{aligned} T^{(-1)}_\alpha \propto \int d\varvec{r}\left( r_\alpha r_\beta -2r^2\delta _{\alpha \beta }\right) \nabla ^2j_\beta \propto D_\alpha \ , \end{aligned}$$

since $\nabla ^2\left( r_\alpha r_\beta -2r^2\delta _{\alpha \beta }\right) =-10\delta _{\alpha \beta }$. This invites one to conclude that the electric dipole moment not only is the actual “parent“ term of the series of mean-square radii here, but also is the lowest order term of the toroidal multipole family. Note also that for the mean-square radii of any familiar electric or magnetic multipole the above procedure cannot extended beyond $n = 0$, yielding vanishing result for $n=-1$. Indeed, consider, for example, an electric quadrupole – the *r*-dependent weight in the integrand satisfies $\nabla ^2(3r_\alpha r_\beta -r^2\delta _{\alpha \beta })=0$. So, despite the fact that the charge and toroidal multipoles are independent families their lowest orders seem to agree.

To show that this coincidence is a direct consequence of the delocalized nature of longitudinal currents, as well as to place the above heuristic discussion on a firm ground, we need to define multipole moments precisely. A succinct way to do so is to introduce the following orthonormal basis of vector fields^15,23^

24 $$\begin{aligned}&\varvec{ F}_{lmk}^{(-)}(\varvec{r})=\frac{\nabla F_{lmk}(\varvec{r})}{ik}\nonumber \ ,\\&\varvec{ F}_{lmk}^{(0)}(\varvec{r})=\frac{\varvec{\nabla }\times \left( \varvec{r} F_{lmk}(\varvec{r})\right) }{-i\sqrt{l(l+1)}}\nonumber \ ,\\&\varvec{ F}_{lmk}^{(+)}(\varvec{r})=\frac{\varvec{\nabla }\times\varvec{\nabla }\times \left( \varvec{r} F_{lmk}(\varvec{r})\right) }{-ik\sqrt{l(l+1)}} \ . \end{aligned}$$

where $F_{lmk}=j_l(kr)Y_{lm}(\varvec{n})$, $j_l$ is the Bessel function and $Y_{lm}$ are the spherical harmonics. Their origin and properties are explained in Supplementary Information, Appendix D. For our purposes it is most important to note that the so-called form-factors^15,21,25,26^

25 $$\begin{aligned} m^{(\lambda )}_{lm}(k)=\int d\varvec{r}\, \varvec{j}(\varvec{r}) \cdot \left( \varvec{ F}^{(\lambda )}_{lmk}(\varvec{r})\right) ^* \end{aligned}$$

directly encode both the multiple moments and mean-square radii of a current distribution.

More specifically, form-factors $m^{(0)}_{lm}(k)$ and $m^{(+)}_{lm}(k)$ correspond to magnetic and toroidal multipoles, respectively, while $m^{(-)}_{lm}(k)$ to the charge multipoles, which is clear from the fact that $\varvec{ F}^{(0)}$ and $\varvec{ F}^{(+)}$ are orthogonal to $\varvec{j}_\parallel$. Indices *lm* in each form-factor describe the multipole being probed (dipole, quadrupole etc.), while *k* defines the radial profile of each multipolar mode and corresponds to the mean-square radii.

The multipoles themselves are proportional to form-factors at $k=0$. For instance (up to the usual transformation between the Cartesian and spherical basis)

26 $$\begin{aligned} \varvec{D}\propto m^{(-)}_{1,i}(k=0) \ . \end{aligned}$$

The mean square radii appear as coefficients in the Taylor expansion of $m^{(\lambda )}_{lm}(k)$. For example, the mean-square radii of the dipole moment (21) are proportional to

27 $$\begin{aligned} D^{(n)}\propto \frac{d^{2n}}{dk^{2n}}m^{(-)}_{1, i}(k)\Big |_{k=0} \ . \end{aligned}$$

Now, while form-factors $m^{(\lambda )}_{lm}(k)$ are in general independent functions and describe three independent multipole families, functions $\varvec{ F}_{lmk}^{(-)}$ and $\varvec{ F}_{lmk}^{(+)}$ have the same behavior at small *k*

28 $$\begin{aligned} \varvec{ F}_{lmk}^{(+)}\approx \sqrt{l(l+1)}\varvec{ F}_{lmk}^{(-)},\qquad k\rightarrow 0 \ . \end{aligned}$$

As a consequence, the multipole moments defined by $m^{(-)}_{lm}(0)$ and $m^{(+)}_{lm}(0)$ coincide (up to numerical factors). Crucially though, $m^{(-)}_{lm}(k)$ and $m^{(+)}_{lm}(k)$ cease to agree beyond $k=0$ and, hence, the charge and toroidal mean-square radii are in general all different.

The above analysis formalizes our heuristic derivation at the beginning of this section, which showed that the toroidal dipole moment has the usual electric dipole moment as its “parent multipole“ and that the same applies to all toroidal multiples. Because of this coincidence, the toroidal family is usually defined to start one order higher, i.e. the first mean square radii of form-factors $m^{(+)}_{lm}(k)$ are considered to be the primary toroidal multipoles^15^. In retrospect, it would, perhaps, be more consistent referring to the quantities $m^{(+)}_{lm}(k=0)$ (and thus $\propto m^{(-)}_{lm}(k=0)$) also as toroidal rather than charge multipole moments, because the mean-square radii of the toroidal multipoles all contribute to radiation, while the charge mean-square radii do not^23,24^.

We can now identify two factors, which condition the noted relation between the charge and toroidal multipoles. The first factor is the apparent numerical coincidence stated in Eq. (28). In fact, the form-factor functions (25) are fixed uniquely by the Helmholtz decomposition together with requirements of irreducibility and parity (see Supplementary Information, Appendix D), so this apparent numerical coincidence has deeper roots. The second factor is that although charge multipole moments are originally defined only via the longitudinal currents, while toroidal moments only via transverse currents, the delocalized nature of the former leads to a connection between the two types of currents. Note, in particular, that $k\rightarrow 0$ limit (when the charge and toroidal form-factors coincide) corresponds to the infinite-wavelength limit. In this regime the distinction between $\varvec{j}_\parallel$ and $\varvec{j}_\perp$ becomes negligible, as they only differ in a finite region, which cannot be resolved by an electromagnetic wave with an infinitely large wavelength.

## Conclusions

We have discussed the role of longitudinal currents for radiating spatially localized systems of non-stationary charges in condensed matter and the seeming absence of transverse currents (associated with radiation) in such systems that formally follows from the continuity equation. Our analysis based on the Helmholtz decomposition shows that, in general, longitudinal currents are not spatially confined even when the time-dependent charge densities that generate them are. The most important consequence of this is the implicit appearance of transverse currents (which must also be non-vanishing everywhere in space), even though this cannot be inferred directly from the continuity equation. The demonstrated coexistence of the two kinds of currents and intimate relationship between them in localized systems of radiating charges indicate that longitudinal currents should not be considered completely irrelevant to electromagnetic radiation. The latter is especially true when radiation occurs in partially conducting condensed matter. We have also shown that it is the connection between the two kinds of currents that fuels the ongoing confusion surrounding the charge and toroidal multipoles, which are defined, respectively, via longitudinal and transverse currents.

### Supplementary Information

Supplementary Information.

## Data Availability

All data generated or analysed during this study are included in this published article and its supplementary information file.
