# Supplementary material for: On the role of longitudinal currents in radiating systems of charges
Source: Sci Rep. 2024 Jul 19;14:16666. doi: 10.1038/s41598-024-66848-7 (PMC11271573; doi:10.1038/s41598-024-66848-7)
Supplement: Supplementary file 1 — Supplementary Information. [file 41598_2024_66848_MOESM1_ESM.pdf]

Supplementary Information: On the Role of Longitudinal Currents in Radiating Systems of Charges

Nikita A. Nemkov
Russian Quantum Center, Skolkovo, Moscow 121205, Russia and
National University of Science and Technology MISIS, Moscow 119049, Russia

Vassili A. Fedotov * v.fedotov@skoltech.ru
Centre for Photonic Science and Engineering, Skolkovo Institute of Science and Technology, Moscow 121125, Russia

Appendix A: Maxwell's equations

Here we collect our conventions regarding Maxwell's equations. We work with harmonic time-dependence, frequency is denoted by ω, $k = \omega/c$ is the wavenumber. Maxwell's equations can be written as

$$\mu_0 \boldsymbol{j}(\boldsymbol{r}) = -(k^2 + \nabla^2)\boldsymbol{A}(\boldsymbol{r}) + \boldsymbol{\nabla}(\boldsymbol{\nabla} \cdot \boldsymbol{A}(\boldsymbol{r})) \ , \tag{A1}$$

where $\boldsymbol{A}(\boldsymbol{r})$ is a vector potential in the Weyl gauge (vanishing scalar potential). In this gauge the electric and magnetic fields are related to the vector potential as follows

$$\boldsymbol{E}(\boldsymbol{r}) = ikc\boldsymbol{A}(\boldsymbol{r}), \qquad \boldsymbol{H}(\boldsymbol{r}) = \boldsymbol{\nabla} \times \boldsymbol{A}(\boldsymbol{r}) \ . \tag{A2}$$

Conversely, assuming that the vector potential decays at infinity it can be found by solving (A1)

$$\boldsymbol{A}(\boldsymbol{r}) = \frac{\mu_0}{4\pi k^2} \int d\boldsymbol{r}'\, G(\boldsymbol{r} - \boldsymbol{r}') \left(k^2 \boldsymbol{j}(\boldsymbol{r}') + \boldsymbol{\nabla}(\boldsymbol{\nabla} \cdot \boldsymbol{j}(\boldsymbol{r}'))\right) \ , \tag{A3}$$

where $G(\boldsymbol{r} - \boldsymbol{r}') = \frac{e^{ik|\boldsymbol{r}-\boldsymbol{r}'|}}{|\boldsymbol{r}-\boldsymbol{r}'|}$ is the Green function for Helmholtz equation $(\nabla^2 + k^2)G(\boldsymbol{r}) = -4\pi\delta^{(3)}(\boldsymbol{r})$.

The transverse part of the current satisfies $\boldsymbol{\nabla} \cdot \boldsymbol{j}_\perp = 0$ and gives rise to the transverse part of the vector potential

$$\boldsymbol{A}_\perp(\boldsymbol{r}) = \frac{\mu_0}{4\pi} \int d\boldsymbol{r}'\, G(\boldsymbol{r} - \boldsymbol{r}') \boldsymbol{j}_\perp(\boldsymbol{r}') \ . \tag{A4}$$

The longitudinal part of the current satisfies $\boldsymbol{\nabla}(\boldsymbol{\nabla} \cdot \boldsymbol{j}_\parallel) = \nabla^2 \boldsymbol{j}_\parallel$, hence

$$\boldsymbol{A}_\parallel(\boldsymbol{r}) = -\frac{\mu_0}{k^2} \boldsymbol{j}_\parallel(\boldsymbol{r}) \ . \tag{A5}$$

Outside of a localized source $\boldsymbol{j}_\perp = -\boldsymbol{j}_\parallel = 0$ and, therefore, both $\boldsymbol{A}_\perp$ and $\boldsymbol{A}_\parallel$ can be expressed via $\boldsymbol{j}_\perp$ alone.

Appendix B: Non-radiating charge densities

In the main text we have been careful to make reservations while claiming that any charge density will be consequential for the radiation fields. There is a notable exception. Let us reconsider the Poisson equation (3) and ask whether there exist a charge density ρ_s which actually does not produce a potential ϕ_s (and hence the electric field) outside its domain of definition. The answer is yes, and it is simple to describe all such densities. Assume that ρ_s is a Laplacian of some function ϕ, i.e. $\rho_s = \nabla^2\phi$ and ϕ is zero outside R. Then from (3) it follows that the potential ϕ_s is just equal to ϕ up to constants and hence is itself confined (the argument is almost tautological).

This implies the existence of charge densities that do not produce any electric fields outside. Translated into the language of the Helmholtz decomposition this means that some modifications of the longitudinal current do not affect the transverse part. Namely, one can add to $\boldsymbol{j}$ a term of the form $\boldsymbol{\nabla}\phi$ with any confined function ϕ.

Appendix C: Volume integral of longitudinal currents

Here we show that for a spatially confined current $\boldsymbol{j}$ its longitudinal part $\boldsymbol{j}_{\parallel}$ satisfies

$$\int d\boldsymbol{r}\, \boldsymbol{j}_{\parallel} = 0 \ , \tag{C1}$$

which makes it possible to express all multipole terms as integrals of $\boldsymbol{j}_{\perp}$ alone. As explained in Section I, relation between $\boldsymbol{j}$ and $\boldsymbol{j}_{\parallel}$ arises through a Poisson equation

$$\nabla^2\psi(\boldsymbol{r}) = \boldsymbol{\nabla}\cdot\boldsymbol{j}(\boldsymbol{r}), \qquad \boldsymbol{j}_{\parallel}(\boldsymbol{r}) = \boldsymbol{\nabla}\psi \ , \tag{C2}$$

which is solved by

$$\psi(\boldsymbol{r}) = -4\pi\int d\boldsymbol{r}'\, \frac{\boldsymbol{\nabla}\cdot\boldsymbol{j}(\boldsymbol{r}')}{|\boldsymbol{r}-\boldsymbol{r}'|} \ . \tag{C3}$$

Because the source is spatially confined the range of $\boldsymbol{r}'$ is bounded and for large enough $\boldsymbol{r}$ we can write

$$\psi(\boldsymbol{r}) = -\frac{4\pi}{r}\int d\boldsymbol{r}'\boldsymbol{\nabla}\cdot\boldsymbol{j}(\boldsymbol{r}') + \frac{4\pi}{r^2}\int d\boldsymbol{r}'(\boldsymbol{n}\cdot\boldsymbol{r}')\boldsymbol{\nabla}\cdot\boldsymbol{j}(\boldsymbol{r}') + O\left(\frac{1}{r^3}\right), \tag{C4}$$

where $\boldsymbol{n} = \boldsymbol{r}/r$. The first term here is zero by the Gauss theorem, while the second can be rewritten

$$\psi(\boldsymbol{r}) = \frac{4\pi}{r^2}\boldsymbol{n}\cdot\boldsymbol{V} + O\left(\frac{1}{r^3}\right), \quad \boldsymbol{V} = \int d\boldsymbol{r}'\boldsymbol{r}'(\boldsymbol{\nabla}\cdot\boldsymbol{j}(\boldsymbol{r}')) \ . \tag{C5}$$

Here $\boldsymbol{V}$ is an analog of the dipole moment, but for our purposes this interpretation is not important. $\boldsymbol{V}$ is just a constant vector characterizing current distribution $\boldsymbol{j}$.

Now we are ready to prove (C1). Consider

$$\int d\boldsymbol{r}\, \boldsymbol{j}_{\parallel} = \int d\boldsymbol{r}\, \boldsymbol{\nabla}\psi = \lim_{R\to\infty}\int_{r=R} 4\pi r^2 d\boldsymbol{n}\left(\frac{\boldsymbol{n}\cdot\boldsymbol{V}}{r^2} + O\left(\frac{1}{r^3}\right)\right) \ . \tag{C6}$$

The leading term vanishes due to identity $\int d\boldsymbol{n}\,\boldsymbol{n} = 0$ (the average of the normal vector over a unit sphere is zero), while the subleading terms vanish in the $R\to\infty$ limit. This establishes (C1).

Appendix D: Multipole form-factors

Functions $\boldsymbol{\mathcal{F}}^{(\lambda)}_{lmk}$ introduced in (24) are regular solutions to the Helmholtz equation

$$(\nabla^2 + k^2)\boldsymbol{\mathcal{F}}^{(\lambda)}_{lmk} = 0 \tag{D1}$$

and satisfy orthogonality and completeness relations

$$\begin{aligned} \int d\boldsymbol{r}\, \boldsymbol{\mathcal{F}}^{(\lambda)}_{lmk}(\boldsymbol{r})\cdot\boldsymbol{\mathcal{F}}^{*(\lambda')}_{l'm'k'}(\boldsymbol{r}) &= \frac{(2\pi)^3}{k^2}\delta^{\lambda\lambda'}\delta_{ll'}\delta_{mm'}\delta(k-k') \ , \\ \sum_{lmk\lambda}\left[\boldsymbol{\mathcal{F}}^{(\lambda)}_{lmk}(\boldsymbol{r})\right]_i\left[\boldsymbol{\mathcal{F}}^{*(\lambda)}_{lmk}(\boldsymbol{r}')\right]_j &= (2\pi)^3\delta_{ij}\delta(\boldsymbol{r}-\boldsymbol{r}') \ . \end{aligned} \tag{D2}$$

Under parity transformations $\boldsymbol{x}\to-\boldsymbol{x}$ they behave as

$$\mathcal{F}^{\lambda}_{lmk}(-\boldsymbol{x}) = (-1)^{l+\lambda}\mathcal{F}^{\lambda}_{lmk}(\boldsymbol{x}) \ . \tag{D3}$$

The scalar functions $\mathcal{F}_{lmk} = j_l(kr)Y_{lm}(\boldsymbol{n})$ that are used to define $\boldsymbol{\mathcal{F}}^{(\lambda)}_{lmk}$ also solve the Helmholtz equation and have the following $k\to 0$ asymptotic

$$\mathcal{F}_{lmk}(\boldsymbol{r}) = \frac{4\pi i^l}{(2l+1)!!}(kr)^l Y_{lm}(\boldsymbol{n})(1+O(k)) \ , \tag{D4}$$

from which (28) can be derived.
